# Program assessment of efforts to improve the quality of postpartum counselling in health centers in Morogoro region, Tanzania

**DOI:** 10.1186/s12884-018-1906-y

**Published:** 2018-07-04

**Authors:** Amnesty LeFevre, Rose Mpembeni, Charles Kilewo, Ann Yang, Selena An, Diwakar Mohan, Idda Mosha, Giulia Besana, Chrisostom Lipingu, Jennifer Callaghan-Koru, Marissa Silverman, Peter J. Winch, Asha S. George

**Affiliations:** 1 Department of International Health, Johns Hopkins Bloomberg School of Public Health, Baltimore, MD USA; 2 School of Public Health and Family Medicine, Division of Epidemiology and Biostatistics, University of Cape Town, Cape Town, South Africa; 3 School of Public Health and Social Sciences, Muhimbili University of Health and Allied Sciences, P.O. Box 65015, Dar es Salaam, Tanzania; 4 Department of Obstetrics and Gynaecology, Muhimbili University of Health and Allied Sciences, P.O. Box 65001, Dar es Salaam, Tanzania; 5 Jhpiego Tanzania, Box 9170, Dar es Salaam, PO Tanzania; 6 School of Public Health, University of the Western Cape, Bellville, South Africa

**Keywords:** Postnatal care, Counselling, Postpartum care, Primary health care, Tanzania

## Abstract

**Background:**

The postpartum period represents a critical window where many maternal and child deaths occur. We assess the quality of postpartum care (PPC) as well as efforts to improve service delivery through additional training and supervision in Health Centers (HCs) in Morogoro Region, Tanzania.

**Methods:**

Program implementers purposively selected nine program HCs for assessment with another nine HCs in the region remaining as comparison sites in a non-randomized program evaluation. PPC quality was assessed by examining structural inputs; provider and client profiles; processes (PNC counselling) and outcomes (patient knowledge) through direct observations of equipment, supplies and infrastructure (*n* = 18) and PPC counselling (*n* = 45); client exit interviews (*n* = 41); a provider survey (*n* = 62); and in-depth provider interviews (*n* = 10).

**Results:**

While physical infrastructure, equipment and supplies were comparable across study sites (with water and electricity limitations), program areas had better availability of drugs and commodities. Overall, provider availability was also similar across study sites, with 63% of HCs following staffing norms, 17% of Reproductive and Child Health (RCH) providers absent and 14% of those providing PPC being unqualified to do so. In the program area, a median of 4 of 10 RCH providers received training. Despite training and supervisory inputs to program area HCs, provider and client knowledge of PPC was low and the content of PPC counseling provided limited to 3 of 80 PPC messages in over half the consultations observed. Among women attending PPC, 29 (71%) had delivered in a health facility and sought care a median of 13 days after delivery. Barriers to PPC care seeking included perceptions that PPC was of limited benefit to women and was primarily about child health, geographic distance, gaps in the continuity of care, and harsh facility treatment.

**Conclusions:**

Program training and supervision activities had a modest effect on the quality of PPC. To achieve broader transformation in PPC quality, client perceptions about the value of PPC need to be changed; the content of recommended PPC messages reviewed along with the location for PPC services; gaps in the availability of human resources addressed; and increased provider-client contact encouraged.

**Electronic supplementary material:**

The online version of this article (10.1186/s12884-018-1906-y) contains supplementary material, which is available to authorized users.

## Background

While home to just 12% of the world’s population, 56% of maternal deaths [1] and 50% of deaths in children under five years of age occur in sub-Saharan Africa [2]. Tanzania is the fifth most populous country in sub-Saharan Africa and one of 10 countries globally which together accounted for 59% of all maternal deaths in 2015 [3]. While declines in maternal and child deaths occurred throughout the last four decades, mortality continues to remain unacceptably high. In 2015, for every 100,000 live births, 398 maternal deaths occurred, and among children under 5, for every 1000 live births, 67 deaths occurred [1, 4].

The postpartum period, from birth to six weeks after birth represents a critical window of time where many maternal and child deaths occur – largely due to preventable causes [1, 4]. Postpartum care (PPC) services encompass prevention, early detection and management of complications for mothers and newborns, as well as provision of advice and services on breastfeeding, birth spacing, immunizations, nutrition and HIV services [5]. In Tanzania, the Ministry of Health and Social Welfare (MOHSW) recommends that mothers and children receive a minimum of 4 PPC consultations at the following times: within 48 h of delivery, between 3 and 7 days, 8–28 days, and 29 to 42 days (Table 1) [5, 6]. Despite these recommendations and near universal completion of at least one antenatal care visit (96%), only 50% of deliveries in 2015 occurred in facilities and only 35% of women with a live birth outcome received a PPC checkup following delivery [7].

**Table 1:** Ministry of Health and Social Welfare Guidelines for PPC Services in Tanzania

Timing and recommended service content for routine postpartum care visits	
1. ≤24 h after delivery: • Clinical assessment of mothers and newborns within an hour after birth;	
• Infant feeding practices, skin-to-skin contact, and identification of danger signs for both mother and baby;	
• Prior to facility discharge providers to give counseling on danger signs, nutrition, family planning, self-care for the mother and child at home, as well as preventive measures such as bed nets and a supply of iron and vitamin A to the mother.	
2. ≤7 days after birth: provider follow up with mother and baby about danger signs and continue health education.	
3. 28 days after the birth: child immunization and examinations to check for continued recovery of mother and healthy development of child.	
4. 42 days after the birth: child immunization and examinations to check for continued recovery of mother and healthy development of child.	
5. Reproductive Child Health follow-up care for 1 year postpartum	
6. Maternal and/or baby consultations as needed in event of complications	
Basic PPC Care Package in Tanzania	
Providers should:	
• Discuss breastfeeding, breast care or alternate infant feeding options for HIV-positive women;	
• Emphasize the Lactational Amenorrhoea Method (LAM) and later transition to other family planning methods;	
• Provide nutritional support;	
• Counsel on self-care and other healthy practices;	
• Discuss mother and baby danger signs;	
• Make complication readiness plans;	
• Offer HIV counseling and testing;	
• Provide immunizations;	
• Offer preventive measures (i.e., iron/folate supplements);	
• Provide antiretroviral medicine and cotrimoxazole prophylaxis as needed.	

Increasing access to and utilization of health services is necessary but alone may not be sufficient to improve maternal, newborn and child health (MNCH) [8]. Evidence is emerging that attention must be also placed on the quality of care [9]. In Tanzania, data on service quality are available from Maternal Child Health Integrated Program surveys, however, information specific to PPC counselling is limited [10].

In an effort to improve utilization and quality of facility-based PPC services, the Tanzanian MOHSW, with support from the USAID-funded Mothers and Infants, Safe Healthy, Alive (MAISHA) program facilitated by Jhpiego, conducted a 6-day in-service training of 2–4 health care providers in the Reproductive and Child Health (RCH) wards of nine Health Centers and one Regional Hospital in five districts of Morogoro in 2010 and 2011. In-service training drew upon national PPC guidelines and was complemented by the introduction of MOHSW-approved PPC registers to track service utilization, and quarterly supportive supervision from MOHSW and Jhpiego personnel. We sought to measure the quality of facility PPC and assess the effects of programmatic inputs to improve PPC service delivery in nine Health Centers that received additional training and supervision input with nine comparison Health Centers without such inputs.

### Assessing quality of postpartum care

Frameworks to assess quality of care trace back to Donabedian’s [11] three dimensions: (1) *structure*, or settings within which care takes place, (2) *process* of providing care, and (3) *outcome* of care on health status [12–16]. These dimensions were supplemented by varying perspectives at different levels (care by practitioners, amenities, care undertaken by patients, and care received by community) [14]. The definition of quality was also broadened to include dimensions of effectiveness (delivering evidence-based care that results in improved health outcomes), efficiency (delivering health care in a manner which maximizes resource use and avoids waste), acceptability (care takes into account individual/cultural preferences), and equity (care does not vary unfairly by social characteristics) [12]. In 2006, these dimensions were expanded by WHO to include accessibility (care is timely, geographically reasonable, and provided in a setting where skills and resources are appropriate to medical need) and safety (minimal risk/harm to users) [17–19].

**Fig. 1:**
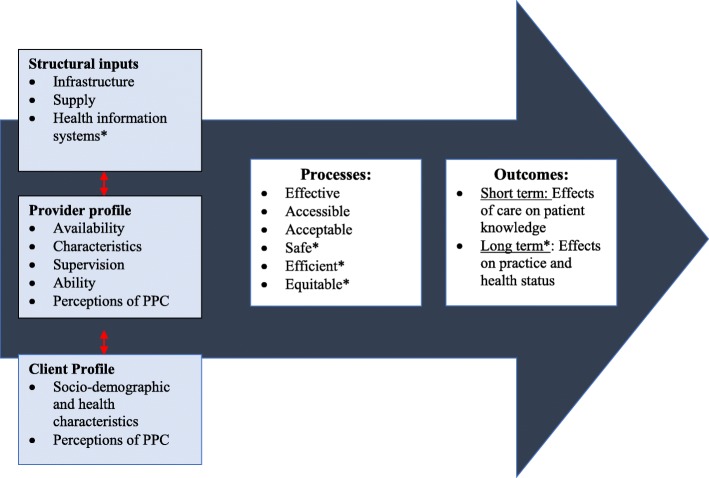
Concepual Framework for assessing the context and Quality of PPC counseling in health centres in Morogoro Tanzania. ***Legend***: *Not measured in the current analysis. ***References***: 1. Donabedian A. The quality of care. How can it be assessed?1988, ARCH Pathol Lab Med 1997, 121 (11):Pg 1145-50. 2. Atherton F. G. Mbekem and I. Nyalusi. Improving service quality from thre Tanzania Family Health Project. Int J Qual Health Care. 3. WHO. Quality of care. A process for making strategic choices in health systems:2006, Worls Health Organization: France 4:p3

Based on this trajectory of work on quality of care, we modified Donabedian’s linear framework of *structure*, *processes* and *outcomes* to include both provider and client perspectives [20]. Figure 1 depicts the relationship between these domains, illustrating that attributes in the service environment (structural inputs) interact with client and provider profiles to influence delivery and receipt of care (processes), which correspond to changes in short- (patient knowledge) and long-term (careseeking and health status) outcomes. Consideration of the dynamic interaction between these dimensions provides a more comprehensive picture of quality of care in health facilities.

## Methods

### Study site, design and sampling

Located 200 km southwest of Dar es Salaam, Morogoro is the sixth most populous and second largest region (2.2 million population dispersed over 70,000 km; 73% in rural areas) of Tanzania’s 30 regions [13]. Careseeking for PPC in Morogoro region (35%) mirrors the national average, while other indicators are slightly higher than national averages: antenatal care utilization (99% versus 98%), facility deliveries (75% versus 63%), and skilled birth attendance (78% versus 64%) [1, 4]. A non-randomized program assessment was carried out in 18 Health Centers (9 program and 9 comparison) located in nine districts (Gairo, Kilosa, Morogoro District Council, Mvomero and Ulanga) in late 2012. All health facilities provide the same core package of MNCH services. However, the nine program HCs were purposively selected by Jhpiego and the District Reproductive and Child Health Coordinator based on their higher utilization of Prevention of mother-to-child transmission services (program activities were funded by USAID using PEPFAR funding). The nine remaining HCs in these were selected for comparison purposes during the program assessment.

To assess quality of PPC services, we examined the following domains: 1. Structural inputs; 2. Provider profiles; 3. Client profiles; 4. Processes, including content of PPC counselling and accessibility; and 5. Outcomes, including short-term effects of care on patient knowledge (Fig. 1).

Structural inputs, including availability of PPC infrastructure, drugs, equipment, supplies, and testing services, were assessed through direct observations in all 18 HCs using standardized checklists of indicators drawn from the Service Provision Assessment tools [4] and the Maternal Child Health Integrated Program quality of care toolkit [21]. In the event of drug ‘stock-outs,’ the HC in-charge and/or attending pharmacist were asked to provide details on the duration and/or ever-availability of the product in question. Data on the reported frequency of supervision were collected during provider interviews and stratified by source and content.

PPC providers were assessed according to their availability, knowledge, social profile, and perceptions of PPC. PPC health provider availability was determined through interviews with facility in-charges and compared with national guidelines. In addition, a structured questionnaire was administered to the sub-sample of RCH providers available during the research team’s visit. Knowledge was assessed across five major domains: (i) postnatal danger signs, (ii) essential newborn care and care for low birth weight infants, (iii) maternal hygiene and recovery, (iv) infant and maternal nutrition, (v) family planning, HIV/ AIDS, and malaria. Data was drawn from the subset of RCH health workers who reported providing PPC services in the preceding seven days.

Client profile, including demographic and personal/family characteristics, maternal health background, and PPC perceptions were assessed through exit interviews with PPC counselling recipients. Exit interviews included a quantitative survey to assess knowledge, demographic characteristics, travel and waiting times as well as semi-structured in-depth interview questions to capture perceptions of PPC.

Observations of the content of PPC counselling by data collectors during their two days visit of HCs were used to evaluate processes associated with giving care. Content of PPC counselling was evaluated using checklists containing over 85 group and individual counselling messages as per government guidelines. Additional data on job aids use and client/provider interaction were also collected. Longer-term effects of PPC on practice and health status fell beyond the scope of the current analysis (Table 2).

**Table 2:** PPC Data sources

Domains of Quality		Measurement Methods	Sampling	Final sample
Structural inputs	Supply and infrastructure availability	Observations of infrastructure, drugs, supplies, and commodities	100% of all health centers in 5 districts	18 health centers: 9 program, 9 comparison
	Supervision	PPC provider survey	• Interview with facility in-charge to determine available human resources (*n* = 18) • Sub-sample health center RCH providers (*n* = 88) available on day of visit • Sub-analysis of RCH service providers that reported providing PPC services in the preceding 7 days	18 health centers in-charges: 9 program, 9 comparison 62 PPC providers; 38 program area, 24 comparison
Provider profile	Health provider ability and availability			
	Perceptions of PPC	Qualitative in-depth provider interviews	Sub-analysis of PPC providers interviewed as part of qualitative in-depth interviews carried out among 57 RCH providers (mean of 3 per facility)	10 PPC Providers; 6 program area, 4 comparison
Client profile	• Social profile • Health profile	• Qualitative in-depth interviews • PPC client exit interviews	Of 45 PPC clients observed, 41 consented to exit interviews.	41 completed exit interviews; 25 program area, 16 comparison
Process	• PPC Counseling content • PPC Service accessibility and efficiency	Observations of PPC counseling sessions delivered	• Total approved target sample of 240 to detect a 30% difference (design effect of 2.0) in frequency of observed messages on postpartum family planning across study arms. Quota based on availability on day of visit. • 55 PPC Clients presented for services during 36 days of observation (2 days per facility). • Of 55 total PPC clients, 45 consented to observation; 9 departed prior to service delivery and 1 declined consent.	45 completed observations; 26 program area, 19 comparison
Outcome	Client knowledge	Quantitative PPC client exit interviews	Of 45 PPC clients observed, 41 consented to exit interviews.	41 completed exit interviews; 25 program area, 16 comparison

### Data collection

A team of six research assistants, including two social scientists, two medical doctors, and two quantitative researchers, received training from MUHAS and Johns Hopkins School of Public Health (JHSPH) faculty over a one-week period in mid-September 2012. Training included two days of pilot testing in two HCs on the outskirts of Dar es Salaam and was immediately followed by the start of data collection in late September.

In Morogoro, data were collected over a two-day period in each HC. Prior to the start of data collection, study personnel visited the HC in-charges to brief them on data collection objectives, ascertain the days PPC services were provided, and coordinate the timing for data collection. Data collection included direct observations of equipment/supplies, observations of PPC counseling, and provider and client interviews. Observations of PPC counseling were carried out during outpatient clinical service provision – characteristically between 8 am and 1 pm – or at the time of patient discharge (a critical window for PPC counselling) among all consenting PPC clients during the two-day data collection window in each HC. PPC clients were approached prior to the start of counselling, and oral consent for observation and exit interview was obtained. Following observations, an exit interview of about one hour in duration was recorded on paper in a private area of the HC.

Quantitative provider interviews were carried out among consenting RCH providers available during the data collection shift of 8 am-6 pm. In each HC, of the approximately 20 total providers on the roster, 5–10 were present during the data collection shift of 8 am-6 pm and of these, 3–5 were RCH providers. Qualitative in-depth interviews were carried out among a sub-sample of about three RCH providers per facility and digitally recorded. Since the focus of this paper was on PPC, analyses focused on the sub-sample of health workers who reported providing PPC services.

### Data analyses

Quantitative data were single entered and cleaned using Epi Info software, with statistical analyses performed using Stata 12.0 and 13.0. A target sample of 180 PPC observations was required to observe a 30% difference in the content of counselling on newborn danger signs between study arms. The total sample achieved of 45 PPC observations fell short of this, and thus descriptive analyses were carried out using basic cross-tabulations. For facility readiness and counselling content, summary composite scores were calculated by the probability-weighted average for each domain. To look for associations at the individual level in the duration of counselling and the scores for the different domains of counselling, we used non–parametric correlation methods (Spearman’s rho).The Spearman statistic makes adjustments for ties by averaging ranks, with values ranging from − 1 (perfect negative association) to + 1 (perfect positive association) and with zero indicating the absence of association.

Qualitative elements of client exit interviews were recorded by hand, translated during data collection and coded manually, while the qualitative elements of provider interviews were recorded electronically, transcribed and translated into English, before being coded using Atlas.ti. Codes derived from the structure of the interview guide and from themes that emerged from daily, midpoint and endpoint team debriefings. Draft write-ups of the thematic analysis were circulated among co-investigators before finalization.

Preliminary findings from both the quantitative and qualitative analysis were shared with key decision makers in Tanzania belonging to the MOHSW and Jhpiego for their feedback and review prior to publications being drafted.

## Results

### Structural inputs

**Fig. 2:**
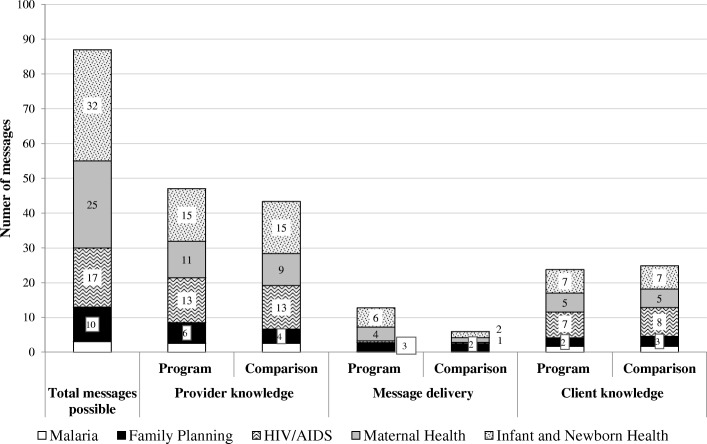
Availability of essential PPC infrastructure, drugs, equipment, supplies and testings services in 18 health centres in Morogoro region, Tanzania in 2012: Mean composite scores. ***Legend:*** ***Physical Infrastructure*** (On call staff housing, electricity, water source, client toilet, waiting area, wash basin with running water in the PPC area). ***Drugs and Commodities***: (Iron/folic acid, vitamin A, tetracycline, polio vaccine, BCG vaccine, DPT vaccine, Condoms, Oral Contraceptive pills, depo provera, intra uterine devices, implants, cotrimoxazole, NVP for mother, NVP for child, AZT, 3TC, ARVs.) ***Equipment /supplies:*** (Tape measure, sterile clamps, thermometer, baby weighing scale, sterile gloves, syringes, vaccine thermometer, ice box, ice packs and fridge). ***Diagnostic tests:*** (CD4 count, HIV testing, DBS HIV for newborns)

Physical infrastructure, equipment and supplies were comparable across program and comparison HCs (Fig. 2). An estimated 39% of facilities were observed to not have a functioning water source and 33% had no electricity in the newborn care/labor delivery ward. In qualitative interviews, across all HCs providers discussed the challenges imposed by limited space, particularly when juggling the needs of different MCH services, including ANC and PPC.

Program HCs were observed to have better availability of drugs, commodities and testing supplies for PPC (Fig. 2), including CD4 (89% versus 56%), adult HIV testing kits (78% versus 56%), early infant HIV diagnosis (89% versus 78%) and hemoglobin (89% versus 56%). The availability of HIV commodities was similar across both program and comparison HCs. Cotrimoxazole was observed in all HCs; however, stock-outs of Nevirapine (single dose and syrup), Zidovudine, and Lamivudine were noted in more than 25% of all HCs. Among family planning commodities, program HCs were observed to have a wider selection of contraceptives in stock (average of 4.9 of 6 total products versus 3.3 in comparison HCs).

### Provider profiles

HCs were staffed by a median of 24 (range 11–54) providers, nearly half of whom (11, range of 3–24) reported providing MNCH services (Table 3). Only 63% of HCs met MOHSW recommended staffing requirements for MNCH cadres - Registered Nurses, Enrolled Nurses, and Medical Attendants. In addition, among the total MNCH providers listed on the rosters, 17% were reported by the HC In-Charge to be absent. Shortages in staffing, as a result of misallocation of staff and absenteeism, were reported by providers to make responding to diverse client needs challenging.

*“But still the challenge is that there are few attendants…maybe at RCH perhaps people have arrived, maybe there are four for postpartum care, you need to thoroughly examine these [patients] and still there are pregnant women [coming for ANC visits]…so there comes the challenge to observe every aspect [of PPC].” (Registered nurse, 10 years of experience, PPC training)*

**Table 3:** Characteristics of PPC providers interviewed (*n* = 65) and trained (*n* = 19) in 18 health centers in 4 districts of Morogoro region, Tanzania in 2012

	Total	Program	Control
All facility characteristics^a^			
Median number of providers reported per facility	24	28	21
Median number of providers reported providing MNCH per facility	11	10	11
Median number of providers trained in PPC per facility	1	4	0
Characteristics of interviewed RCH providers who reported providing PPC	(*N* = 63)	(*N* = 37)	(*N* = 26)
Female provider	49 (77%)	28 (76%)	21 (79%)
Designation			
Enrolled Nurse	32 (51%)	19 (50%)	15 (58%)
Registered Nurse	10 (16%)	6 (16%)	4 (15%)
Medical Attendant	9 (14%)	7 (18%)	2 (8%)
Clinical officer/ Ass. Clinical officer	5 (8%)	3 (8%)	2 (8%)
Other^a^	6 (10%)	3 (8%)	3 (12%)
Years working as a health worker (Median)	13	13	12.5
Years working in this health facility (Median)	4	3	4.5
PPC providers whom have received PPC training	18 (29%)	17 (46%)	1 (4%)
Characteristics of interviewed RCH providers trained in PPC	(*N* = 19)	(*N* = 17)	*(N)* = 2
Female provider	16 (84%)	14 (82%)	2 (100%)
Designation			
Enrolled Nurse	14 (74%)	12 (71%)	2 (100%)
Registered Nurse	4 (21%)	4 (24%)	0%
Other^b^	1 (5%)	1 (6%)	0%
Years working as a health worker (Median)	17	15	29
Years working in this health facility (Median)	4.3	4	15

^a^Data obtained through facility in-charge interview

^b^Other here includes Assistant Medical Officer, Health Officer, and Health Assistant

Among available RCH providers, nearly one-third reported providing PPC services to mothers, children, or both in the preceding seven days (Table 3). PPC providers included Enrolled Nurses (51%), Registered Nurses (16%), and Medical Attendants (14%) – a cadre of providers with limited to no clinical training. The majority of PPC providers were females (77%) who had been working in their current HC for a median of 4.3 years, and as a health care provider for 17 years.

Interviewed providers in both program and comparison areas were asked about their ability to provide PPC. Most felt that they had limited practice in providing PPC as a stand-alone service, as most women were seen soon after a facility delivery and few returned after that.

*“We were studying it at school, but after studying it, practicing it here in working stations was so minimal, only maybe there at the labor room after delivery after they have delivered.” (Enrolled nurse, 5 years of experience, no PPC training)*

PPC training was provided to 4 of 10 (40%) of RCH providers in program HCs. Those trained were primarily female (82%) and enrolled (71%) or registered (24%) nurses. Nearly half of providers interviewed across all HCs reported receiving supervision visits at least once in the 12 months preceding the survey (Additional file 1: Table S1). While all providers interviewed received supervision from Child Health Management Teams (CHMT), regional and MoH supervisors equally, program areas received twice as much supervision from a non-government organization (NGO) (64% vs. 33%) and from district non-Child Health Management Teams (CHMT) (42% vs. 21%) than comparison areas.

**Fig. 3:**
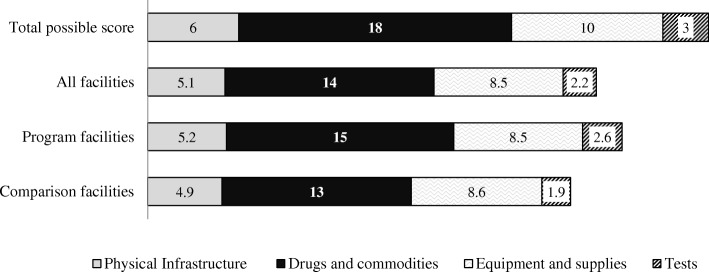
Composite scores of provider knowledge (*N* = 62), observed message delivery (*N* = 45), and client knowledge (*N* = 41) of PPC messages in 18 health centers in 4 districts of Morogoro region, Tanzania in 2012

Despite the training and supervision inputs provided to program areas, wide differences in provider knowledge of PPC counselling messages were not observed across HCs (Additional file 2: Table S2). The proportion of messages that providers reported knowing for each domain was lower for infant, newborn and maternal health, than for HIV, family planning and malaria, (Fig. 3). This waspartly related to the total number of messages to recall for each domain: infant and newborn health (32), maternal health (25), HIV (17), family planning (10) and malaria (3).

### Client profiles

PPC women (26 women in program HCs, 19 women in comparison HCs) were between 20 and 35 years of age (median 24), with limited (48%) to no formal education (33%), mostly facility births (71%), uncomplicated vaginal deliveries (93%), and with deliveries one week prior to the interview (median 7 days) (Table 4).

**Table 4:** Characteristics of PPC counseling sessions observed (n = 45) and clients interviewed (*n* = 41) in 18 health centers in 4 districts of Morogoro region, Tanzania in 2012

	Total	Program	Comparison
PPC Client Characteristics	*N* = 41	*N* = 25	*N* = 16
Age (median time in years)	24	23	28
Health facility delivery	29 (71%)	14 (55%)	12 (76%)
Days since delivery (median)	7.0	7.0	11
Mode of delivery			
Normal without episiotomy	38 (93%)	23 (92%)	15 (94%)
Normal with episiotomy	2 (5%)	1 (4%)	1 (6%)
Cesarean	1 (2%)	1 (4%)	0 (0%)
Years of Schooling			
No formal education	14 (33%)	12 (48%)	2 (12%)
Primary	20 (48%)	10 (40%)	9 (59%)
Secondary	6 (14%)	3 (12%)	3 (18%)
Others	1 (2%)	0 (0%)	2 (12%)
Number of times pregnant prior to delivery of last child (median)	1.5	1.0	2.0
Patient recalled visit characteristics	*N* = 41	*N* = 25	*N* = 16
Duration of travel time to get health facility (median time in minutes)	30	60	15
Duration of waiting time between arriving at clinic and being seen by provider (median time in minutes)	30	30	15
Client perceptions on waiting time			
Long / too long	17 (41%)	11 (43%)	6 (40%)
Acceptable	22 (53%)	13 (52%)	8 (53%)
Short	2 (6%)	1 (5%)	1 (7%)
Reason for PPC Visit			
Baby well-visit	25 (61%)	14 (56%)	11 (69%)
Mother well-visit	14 (34%)	11 (44%)	3 (19%)
Baby symptoms/ sickness	2 (5%)	2 (8%)	0 (0%)
Mother symptoms/ sickness	1 (2%)	1 (4%)	0 (0%)
Observed visit characteristics	*N* = 45	*N* = 26	*N* = 19
Duration of Individual PPC counseling (median time in minutes)	9.5	20	2.0
Counselling type			
Group	12 (30%)	5 (21%)	7 (44%)
Individual	14 (35%)	14 (54%)	1 (6%)
Both	14 (35%)	6 (25%)	8 (50%)
PPC visit number			
At discharge post delivery	9 (22%)	7 (27%)	3 (16%)
First follow up visit after delivery (within 7 days)	20 (49%)	14 (54%)	7 (42%)
Second visit (within 28 days)	9 (22%)	4 (15%)	5 (32%)
Third visit (within 42 days)	12 (29%)	1 (4%)	2 (11%)
PPC Visit recipient			
Child	1 (2%)	1 (4%)	0 (0%)
Mother	13 (31%)	1 (4%)	11 (68%)
Mother and child	27 (67%)	23 (92%)	5 (32%)
Provider used job aides	12 (29%)	1 (4%)	10 (63%)
Provider told client return visit date			
Sets a date for the next visit with the client	41 (100%)	25 (100%)	16 (100%)
Encourages her to return to the next planned visit	40 (98%)	24 (96%)	16 (100%)
PPC provider was the same as that who attended you during a previous visit to this health facility	11 (26%)	6 (23%)	5 (29%)

Women sought PPC services because they felt that PPC was beneficial to ensure child health, particularly with regard to vaccinations and/or receipt of registration cards. Some women named specific diseases against which children are routinely immunized, including polio and tetanus. One woman emphasized the importance of PPC for her child over and above her own care:

*“If I come here and I don’t get any service for myself, I won’t care. But if my child won’t be given immunization or nothing is done to her, I will question.” (28 years old, 15 days since delivery, PPC2)*

PPC for women was largely seen as unnecessary, especially among women with prior healthy, complication-free pregnancy and delivery experiences that did not require them to return to a health facility. The few women that did mention seeking PPC services for themselves, did so largely for family planning. Women noted that they knew family planning would be available and, in some cases, reported being informed by providers to return for family planning counselling and supplies.

*“Some of them [mothers], they come for further mother health examination, but honestly speaking, most of them they don’t know if a mother should also be involved.” (28 years old, 15 days since delivery, PPC2)*

In addition, work demands and other domestic priorities also took precedence for women who had recently given birth. One woman commented:

*“Other women do not come because they do not prioritize. They would rather do their chores at home than come for a clinic visit. She sees that her work is more important than the clinic visit.” (29 years old, 14 days since delivery, PPC2)*

### Processes

In the program area, PPC consultations were a median of 20 min in duration as compared to two minutes in the comparison area. A Spearman’s correlation coefficient determined that the number of messages observed during counselling was significantly associated with the total duration of PPC consultation (rho = 0.44, 95% CI: 0.17 to 0.6) (Table 5).

**Table 5:** Correlation between duration of PPC counselling and message delivery: Composite scores for observed PPC counselling (*n* = 45) in 18 health centers in 4 districts of Morogoro region, Tanzania in 2012. One a scale of 1 to −1, where 1 denotes strong positive correlation and − 1 strong negative correlation, findings suggest that the more time spent on counselling corresponds to more messages delivered

Domain	Total (*n* = 45)	Program (*n* = 26)	Comparison (*n* = 19)
Total Composite Score	0.44 (0.17, 0.65)*	0.46 (0.08, 0.72)*	0.27 (−0.21, 0.64)
Maternal Health	0.56 (0.31, 0.73)*	0.52(0.15, 0.76)*	0.48 (0.04, 0.77)*
Infant & child health	0.41 (0.13, 0.63)*	0.22(− 0.19, 0.57)	0.46(0.01, 0.76)*
HIV	0.27(− 0.03, 0.53)	0.1(− 0.31, 0.47)	0.37 (− 0.1, 0.71)
Family Planning	0.02 (− 0.28, 0.32)	0.51 (0.14, 0.75)*	− 0.34 (− 0.69, 0.14)

*Significant at *p* < 0.05

Additional file 2: Table S2 summarizes the frequency of PPC messages observed to be delivered and Fig. 3 depicts mean composite scores of observed PPC message delivery during individual counselling sessions (*n* = 45) in program and comparison areas. While infant, newborn and maternal health form the bulk of counselling messages delivered, the content delivered is still very limited. While this is somewhat higher in program areas, the frequency of observed messages fell below 25% for all domains assessed (Fig. 3).

In contrast to the low frequency of delivery of PPC messages, 100% of providers were observed to set a date for the next visit and 98% encouraged the woman to return for this scheduled visit.

PPC women reported traveling for a median of 30 min to get to the health facility and waiting for a median of 30 min prior to being seen by a provider. Cumulative travel (to and from), waiting and consultation time was an estimated 209 min for program area PPC women, as compared to 100 min in the comparison area. Women’s perceptions of whether the time taken was acceptable (53%), too long (41%), or too short (6%) were the same in both program or comparison areas (Table 4).

Across program and comparison areas, geographic accessibility, measured by distance and the availability of transport, was cited frequently by women as a reason why women do not attend PPC services.

In addition, women’s access to PPC was also hampered by gaps in the continuity of care. One woman noted that if a pregnant woman did not complete ANC and/or delivered at home, access to information about services after delivery might be limited.

*“Many don’t come for ANC visit and they deliver at home, so they don’t get a chance to know the importance of PPC visits.” (23 years old, 21 days since delivery, PPC3)*

PPC users also reported that when women who delivered at home do reach services, they were often treated harshly by providers and/or asked to pay out of pocket costs for PPC services.

*“Sometimes, those who have delivered at home, they are mistreated…when they come for PPC and they have to pay some amount for them to start PPC visits. For example, I paid 3000 shillings so that I could get a card to start PPC services for myself and my child.”* *(23 years old, 21 days since delivery, PPC3)*

*“…saying the truth when they come here… We blame them, why did she decide to give birth at home.” (Enrolled nurse, 3 years experience, no PPC training)*

### Outcomes

Across program and comparison areas, exit interviews of PPC women following observations (*n* = 41) indicate that over 20% of users are able to recall (unprompted) at least one key message for each of the 10 domains (Fig. 3, Additional file 2: Table S2). Among critical danger signs, women recalled nearly half the 13 newborn danger signs assessed, including fever (55%), difficulty in breastfeeding (43%), pitched cry/irritability (40%), convulsions/fits (30%), and hypo/hyperthermia (20%). Similar trends were observed in women’s recall for maternal danger signs, maternal/infant nutrition, and family planning. While counselling messages for HIV/AIDS were infrequently observed (< 5% for most indicators), women’s knowledge of modalities of transmission, prevention and testing were moderate (32%) to high (64%) (Additional file 2: Table S2).

## Discussion

Our study findings indicate that program HCs were better than comparison HCs with respect to availability of PPC drugs, commodities and testing supplies; reported higher PPC supervision and training; and higher duration and more comprehensive PPC counselling sessions. However, across both areas, physical infrastructure, equipment and supplies were similar, with notable limitations related to water supply and electricity. Deficits in human resources, attributed to poor staffing levels and absenteeism, contributed to the use of unqualified providers and were reported to hinder PPC service delivery across both areas.

The majority of PPC recipients were women with uncomplicated vaginal deliveries (93%) in a health facility (71%). This suggests that women most at risk, including those with complications or who deliver at home may not be seeking PPC. Across both program and comparison areas, PPC services were perceived by clients to be beneficial for child health, while services for women were largely seen as unnecessary. Findings on the overall content of observed counselling sessions, while focused on maternal and child health, were still far below program guidelines.

Evidence on the quality of PPC in low resource settings is emerging [22]. Qualitative interviews in Ghana with providers and women [9, 23, 24] and elsewhere in Tanzania with providers [23], first time fathers [25] and mothers [26], and broader support structures [27, 28] echo findings observed here. This suggests that PPC is not well recognized by women [29] and its value largely tied to the receipt of technical interventions, including immunizations for children and family planning for women. Elsewhere in sub-Saharan Africa, despite higher rates of reported PPC awareness, utilization also remains low [23]. To increase demand, women need to respected by providers and see value in PPC. In addition, efforts to increase availability and quality of PPC services at facility and community levels include introduction of PPC home visits, strengthening postpartum outreach services, integration of PPC with mother and child immunization clinics, distribution of PPC guidelines among health workers and upgrading PPC knowledge and skills through training [30].

While the program sought to improve PPC capacity through provider training and ongoing supportive supervision, only a median of 4 RCH providers per program HC received PPC training. Transparent criteria for selecting providers for training; a planned approach for building capacity among the remaining providers not selected; addressing transport and funding constraints that impede the frequency of supervision visits; and ensuring that providers have sufficient PPC women with which they can maintain their skills is critical to ensure that these training and supervision inputs translate into improved service delivery.

There is discordance between provider knowledge and observed delivery of counselling content across all major domains. This suggests that training and supervisory inputs alone may not be sufficient to improve the content of counselling. Increased duration of PPC consultations corresponded to more counselling messages delivered. While this prolonged client waiting times, women across program and comparison HCs reported similar satisfaction levels. Unless efforts are made to address critical deficits in the availability of human resources (40% of HCs fell short of the minimum MOHSW standard for human resources); absenteeism (17% among MNCH providers); and the use of unqualified providers (medical attendants comprised 14% of PPC service providers) improvements in PPC service delivery are unlikely.

Prioritization of the over 80 PPC messages recommended by national PPC guidelines may ensure that women receive and retain the most critical messages. Increased delivery of core maternal health messages may also serve to overcome perceptions that PPC is for child health and of limited benefit to the mother. Finally, wider dialogue on what services should be delivered optimally at different levels of the health system is needed. Current activities underway by the MOHSW to support a national cadre of community health workers may provide a forum for PPC promotion and counselling at community level; allowing facility based cadres of providers to focus more on delivery of services requiring greater clinical or technical expertise. Further review of and inputs to services at dispensary level may provide a similar opportunity to improve and expand availability of PPC services.

### Limitations

Studies on the quality of PPC conducted to date have largely gathered data through qualitative interviews with providers and/or women. Ours is one of the few studies to triangulate data from multiple sources using qualitative and quantitative methods including direct observations of facilities and service delivery, as well as provider and women’s interviews. Despite these strengths, efforts to determine the quality of PPC were hampered by the infrequency of PPC service utilization and logistical challenges associated with achieving the sample size required to draw statistical inferences. Similarly the small numbers of trained providers meant that assessing knowledge and service delivery comparisons within facilities by training status would not be meaningful. Efforts to assess the differential quality of PPC were additionally limited by the purposive selection of HCs in a non-randomized design by program implementors. The program assessment was not commissioned until two years after the start of the program and thus modifications to the program design were not possible. Elsewhere, PPC quality of care assessments have assumed a pre/post study design [30] and thus better isolated the effect of training and program support activities.

The framework used in this analysis focused on the Health Center level. Inputs from the Regional or District level were not considered and processes related to improvements in efficiency not measured. The latter, defined by WHO as delivering health care in a manner which maximizes resource use and avoids waste [31], would best be measured through economic evaluation or assessments of inputs over outputs using Data Envelopment Analysis [20] or Frontier Analyses. These, along with measurement of health outcomes and impact, were all considered beyond the scope of this analysis.

## Conclusions

Our study measured the quality of PPC and assessed the effects of program efforts at HC level to implement national PPC guidelines, and upgrade provider PPC knowledge and skills through PPC training and supervision in rural Tanzania. Study findings highlight deficits in the quality of PPC services in HCs in five districts of Morogoro region, Tanzania. While viewed favorably for ensuring child health, PPC services were not part of the women’s conception of care as needed for themselves after delivery. Content of care was largely constrained to a few counselling messages on immunizations for newborns, benefits and sources of family planning. While this study is limited by the small sample size of observed PPC clients, findings demonstrate that PPC training and supervision inputs had modest effects on the content of counselling services but did yield improvements in the observed duration of consultation—a factor significantly associated with increased message delivery. Although increased provider-woman contact is needed to improve quality in counselling, these improvements are unlikely unless human resource shortages are addressed, provider capacity built, challenges with respectful maternity care addressed, and efforts to strengthen other levels of the health care system undertaken.

## Additional files

Additional file 1: **Table S1.** Reported supervision provided to RCH providers (*n* = 88) from July 2011 to June 2012. (DOCX 31 kb)

Additional file 2: **Table S2.** Provider knowledge (*N* = 62), delivery (*N* = 45) and client knowledge (*N* = 41) of PPC messages in 18 health centers in 4 districts of Morogoro region, Tanzania in 2012. (DOCX 83 kb)

## Abbreviations

ANC: Antenatal care
HC: Health Center
IEC: Information, education, communication
IP: Integrated Program
JHU: Johns Hopkins Bloomberg School of Public Health
MCHIP: Maternal and Child Health Integrated Program
MEP: Morogoro Evaluation Project
MNH: Maternal, newborn, health.
MOHSW: Ministry of Health and Social Welfare
MUHAS: Muhimbili University of Health and Allied Sciences
PMTCT: Prevention of mother to child transmission
PPC: Postpartum care
PPFP: Postpartum family planning
RCH: Reproductive child health
SSA: Sub-Saharan Africa
