# Supplementary material for: Program assessment of efforts to improve the quality of postpartum counselling in health centers in Morogoro region, Tanzania
Source: BMC Pregnancy Childbirth. 2018 Jul 4;18:282. doi: 10.1186/s12884-018-1906-y (PMC6031177; doi:10.1186/s12884-018-1906-y)
Supplement: Supplementary file 1 — Table S1. Reported supervision provided to RCH providers (n = 88) from July 2011 to June 2012. (DOCX 31 kb) [file 12884_2018_1906_MOESM1_ESM.docx]

**Table S1.** Reported supervision provided to RCH providers (n=88) from July 2011 to June 2012

|  | **Total** | | **Program** | | **Comparison** | |
| --- | --- | --- | --- | --- | --- | --- |
| **Proportion of providers who reported receiving any supervision by source** | | | | | | |
|  | **n=** | **88** | **n=** | **46** | **n=** | **42** |
| NGO members | 43 | 49% | 29 | 64% | 14 | 33% |
| CHMT | 43 | 49% | 21 | 46% | 22 | 52% |
| Regional supervisors | 36 | 41% | 19 | 42% | 17 | 41% |
| MOH supervisors | 33 | 38% | 17 | 38% | 16 | 38% |
| District non CHMT | 28 | 32% | 19 | 42% | 9 | 21% |
| **Focus of NGO supervision visits** | | | | | | |
|  | **n=** | **43** | **n=** | **29** | **n=** | **14** |
| PPC | 14 | 33% | 13 | 45% | 1 | 7% |
| HIV/ AIDS/ PMTCT | 13 | 30% | 10 | 36% | 0 | 28% |
| ANC | 7 | 16% | 6 | 21% | 0 | 7% |
| Family Planning | 2 | 5% | - | 0% | 0 | 14% |
| Delivery | 3 | 7% | 3 | 10% | 0 | 0% |
| Other | 11 | 26% | 10 | 36% | - | 21% |
| **Content of PPC supervision visits** | | | | | | |
|  | **n=** | **14** | **n=** | **13** | **n=** | **1** |
| Checked registers | 11 | 79% | 10 | 77% | 1 | 100% |
| Asked knowledge assessment questions | 1 | 64% | 8 | 62% | 1 | 100% |
| Observed patient consultations | 1 | 64% | 9 | 69% | - | 0% |
| Checked attendance | 1 | 57% | 7 | 54% | 1 | 100% |
| Spoke with patients seeking services | 0 | 57% | 8 | 62% | - | 0% |
| Provided feedback on work | 0 | 57% | 7 | 54% | 1 | 100% |
| Provided training | 0 | 43% | 5 | 38% | 1 | 100% |
| Helped fill registers | 0 | 43% | 6 | 46% | - | 0% |
| Brought supplies | 0 | 36% | 4 | 31% | 1 | 100% |
| Checked storage of vaccination | 0 | 29% | 4 | 31% | - | 0% |
| Helped interpret data | 0 | 29% | 4 | 31% | - | 0% |
| Helped to solve problems | 0 | 29% | 4 | 31% | - | 0% |
| Spoke to community members | 0 | 21% | 2 | 15% | 1 | 100% |
| Helped to plan activities/ work | 0 | 14% | 2 | 15% | - | 0% |

|  |  |  |  |  |  |  |
| --- | --- | --- | --- | --- | --- | --- |
|  | **Total** | | **Program** | | **Comparison** | |
| **Proportion of providers who reported receiving any supervision by source** | | | | | | |
|  | **n=** | **88** | **n=** | **46** | **n=** | **42** |
| NGO members | 43 | 49% | 29 | 64% | 14 | 33% |
| CHMT | 43 | 49% | 21 | 46% | 22 | 52% |
| Regional supervisors | 36 | 41% | 19 | 42% | 17 | 41% |
| MOH supervisors | 33 | 38% | 17 | 38% | 16 | 38% |
| District non CHMT | 28 | 32% | 19 | 42% | 9 | 21% |
| **Focus of NGO supervision visits** | | | | | | |
|  | **n=** | **43** | **n=** | **29** | **n=** | **14** |
| PPC | 14 | 33% | 13 | 45% | 1 | 7% |
| HIV/ AIDS/ PMTCT | 13 | 30% | 10 | 36% | 0 | 28% |
| ANC | 7 | 16% | 6 | 21% | 0 | 7% |
| Family Planning | 2 | 5% | - | 0% | 0 | 14% |
| Delivery | 3 | 7% | 3 | 10% | 0 | 0% |
| Other | 11 | 26% | 10 | 36% | - | 21% |
| **Content of PPC supervision visits** | | | | | | |
|  | **n=** | **14** | **n=** | **13** | **n=** | **1** |
| Checked registers | 11 | 79% | 10 | 77% | 1 | 100% |
| Asked knowledge assessment questions | 1 | 64% | 8 | 62% | 1 | 100% |
| Observed patient consultations | 1 | 64% | 9 | 69% | - | 0% |
| Checked attendance | 1 | 57% | 7 | 54% | 1 | 100% |
| Spoke with patients seeking services | 0 | 57% | 8 | 62% | - | 0% |
| Provided feedback on work | 0 | 57% | 7 | 54% | 1 | 100% |
| Provided training | 0 | 43% | 5 | 38% | 1 | 100% |
| Helped fill registers | 0 | 43% | 6 | 46% | - | 0% |
| Brought supplies | 0 | 36% | 4 | 31% | 1 | 100% |
| Checked storage of vaccination | 0 | 29% | 4 | 31% | - | 0% |
| Helped interpret data | 0 | 29% | 4 | 31% | - | 0% |
| Helped to solve problems | 0 | 29% | 4 | 31% | - | 0% |
| Spoke to community members | 0 | 21% | 2 | 15% | 1 | 100% |
| Helped to plan activities/ work | 0 | 14% | 2 | 15% | - | 0% |
