# Supplementary material for: Program assessment of efforts to improve the quality of postpartum counselling in health centers in Morogoro region, Tanzania
Source: BMC Pregnancy Childbirth. 2018 Jul 4;18:282. doi: 10.1186/s12884-018-1906-y (PMC6031177; doi:10.1186/s12884-018-1906-y)
Supplement: Supplementary file 2 — Table S2. Provider knowledge (N = 62), delivery (N = 45) and client knowledge (N = 41) of PPC messages in 18 health centers in 4 districts of Morogoro region, Tanzania in 2012. (DOCX 83 kb) [file 12884_2018_1906_MOESM2_ESM.docx]

**Table S2. Provider knowledge (N=62), delivery (N=45) and client knowledge (N=41) of PPC messages in 18 health centers in 4 districts of Morogoro region, Tanzania in 2012**

|  | **Total** | | | | | | **Program** | | | | | | **Comparison** | | | | | |
| --- | --- | --- | --- | --- | --- | --- | --- | --- | --- | --- | --- | --- | --- | --- | --- | --- | --- | --- |
|  | **Provider knowledge** | | **Delivery** | | **Client knowledge** | | **Provider knowledge** | | **Delivery** | | **Client knowledge** | | **Provider knowledge** | | **Delivery** | | **Client knowledge** | |
|  | n= | 62 | n= | 45 | n= | 41 | n= | 38 | n= | 26 | n= | 24 | n= | 24 | n= | 19 | n= | 16 |
| **Maternal Health** |  |  |  |  |  |  |  |  |  |  |  |  |  |  |  |  |  |  |
| **Danger signs after delivery for the mother** |  |  |  |  |  |  |  |  |  |  |  |  |  |  |  |  |  |  |
| Excessive vaginal bleeding | 51 | (82%) | 10 | (22%) | 25 | (60%) | 30 | (79%) | 9 | (35%) | 13 | (54%) | 21 | (88%) | 1 | (5%) | 11 | (69%) |
| Headache/blurred vision | 20 | (32%) | 8 | (18%) | 7 | (18%) | 14 | (37%) | 7 | (27%) | 5 | (21%) | 6 | (25%) | 1 | (5%) | 2 | (13%) |
| Severe pain in abdomen or genital region | 37 | (60%) | 7 | (16%) | 7 | (18%) | 22 | (58%) | 6 | (23%) | 4 | (17%) | 15 | (63%) | 1 | (5%) | 3 | (19%) |
| Fever | 37 | (60%) | 5 | (11%) | 21 | (50%) | 27 | (71%) | 5 | (19%) | 13 | (54%) | 10 | (42%) | - | (0%) | 7 | (44%) |
| Foul smelling vaginal discharge | 23 | (37%) | 5 | (11%) | 8 | (20%) | 16 | (42%) | 5 | (19%) | 7 | (29%) | 7 | (29%) | - | (0%) | 1 | (6%) |
| Difficulty breathing/fatigue/breathlessness | 24 | (39%) | 3 | (7%) | 3 | (8%) | 17 | (45%) | 3 | (12%) | 2 | (8%) | 7 | (29%) | - | (0%) | 1 | (6%) |
| Convulsions/fits | 23 | (37%) | 3 | (7%) | 2 | (5%) | 16 | (42%) | 3 | (12%) | 2 | (8%) | 7 | (29%) | - | (0%) | - | (0%) |
| Painful breast/bleeding nipple | 13 | (21%) | 3 | (7%) | 4 | (10%) | 7 | (18%) | 3 | (12%) | 3 | (13%) | 6 | (25%) | - | (0%) | 1 | (6%) |
| Scanty urine (oliguria) | 4 | (6%) | 2 | (4%) | - | (0%) | 4 | (11%) | 2 | (8%) | - | (0%) | - | (0%) | - | (0%) | - | (0%) |
| Loss of consciousness | 18 | (29%) | 1 | (2%) | 2 | (5%) | 13 | (34%) | 1 | (4%) | 1 | (4%) | 5 | (21%) | - | (0%) | 1 | (6%) |
| Difficulty breastfeeding | 5 | (8%) | 1 | (2%) | 5 | (13%) | 4 | (11%) | 1 | (4%) | 3 | (13%) | 1 | (4%) | - | (0%) | 2 | (13%) |
| Abnormal behavior (depression, psychosis) | 11 | (18%) | - | (0%) | 1 | (3%) | 7 | (18%) | - | (0%) | 1 | (4%) | 4 | (17%) | - | (0%) | - | (0%) |
| **Counsel on maternal hygiene and recovery** |  |  |  |  |  |  |  |  |  |  |  |  |  |  |  |  |  |  |
| To keep the genital area clean | 57 | (92%) | 14 | (31%) | 25 | (60%) | 35 | (92%) | 11 | (42%) | 14 | (58%) | 22 | (92%) | 3 | (16%) | 10 | (63%) |
| To change sanitary pads/cloth while there is still vaginal discharge | 55 | (89%) | 11 | (24%) | 16 | (38%) | 35 | (92%) | 9 | (35%) | 9 | (38%) | 20 | (83%) | 2 | (11%) | 6 | (38%) |
| To get adequate rest and sleep | 25 | (40%) | 3 | (7%) | 12 | (30%) | 16 | (42%) | 1 | (4%) | 9 | (38%) | 9 | (38%) | 2 | (11%) | 3 | (19%) |
| To wipe the perineal area front to back | 3 | (5%) | - | (0%) | 3 | (8%) | 2 | (5%) | - | (0%) | 3 | (13%) | 1 | (4%) | - | (0%) | - | (0%) |
| **Counseling on maternal nutrition** |  |  | - |  | - |  | - |  | - |  | - |  |  |  | - |  | - |  |
| The important types of foods to eat | 54 | (87%) | 15 | (33%) | 27 | (65%) | 32 | (84%) | 8 | (31%) | 14 | (58%) | 22 | (92%) | 7 | (37%) | 12 | (75%) |
| To take regular meals | 31 | (50%) | 11 | (24%) | 18 | (43%) | 19 | (50%) | 7 | (27%) | 7 | (29%) | 12 | (50%) | 4 | (21%) | 10 | (63%) |
| Counsels about eating food rich in Vit C | 15 | (24%) | 8 | (18%) | 3 | (8%) | 9 | (24%) | 8 | (31%) | 1 | (4%) | 6 | (25%) | - | (0%) | 2 | (13%) |
| To eat a variety of foods at meals | 52 | (84%) | 6 | (13%) | 16 | (40%) | 31 | (82%) | 3 | (12%) | 10 | (42%) | 21 | (88%) | 3 | (16%) | 6 | (38%) |
| Encourage eating 3 meals and a snack/bite in between meals every day | 10 | (16%) | 3 | (7%) | 4 | (10%) | 7 | (18%) | 2 | (8%) | 3 | (13%) | 3 | (13%) | 1 | (5%) | 1 | (6%) |
| To take 2-3 litres of water a day | 20 | (32%) | 3 | (7%) | 4 | (10%) | 15 | (39%) | 3 | (12%) | 2 | (8%) | 5 | (21%) | - | (0%) | 2 | (13%) |
| Counsels against taking tea, coffee and cola drinks when consuming vitamin C | 3 | (5%) | 1 | (2%) | - | (0%) | 2 | (5%) | 1 | (4%) | - | (0%) | 1 | (4%) | - | (0%) | - | (0%) |
| Encourages woman to cook with iodized salt | 5 | (8%) | - | (0%) | - | (0%) | 4 | (11%) | - | (0%) | - | (0%) | 1 | (4%) | - | (0%) | - | (0%) |
| **Newborn Health** |  |  |  |  |  |  |  |  |  |  |  |  |  |  |  |  |  |  |
| **Danger signs after delivery for the newborn** |  |  | - |  | - |  | - |  | - |  | - |  | - |  | - |  | - |  |
| Redness, bleeding, discharge from the umbilicus | 43 | (69%) | 14 | (31%) | 5 | (13%) | 26 | (68%) | 12 | (46%) | 4 | (17%) | 17 | (71%) | 2 | (11%) | 1 | (6%) |
| Difficulty feeding/ unable to suckle/ Failure to breastfeed | 55 | (89%) | 11 | (24%) | 18 | (43%) | 35 | (92%) | 11 | (42%) | 11 | (46%) | 20 | (83%) | - | (0%) | 6 | (38%) |
| Jaundice/yellow color of the skin and eyes | 24 | (39%) | 9 | (20%) | 2 | (5%) | 15 | (39%) | 9 | (35%) | 1 | (4%) | 9 | (38%) | - | (0%) | 1 | (6%) |
| Fever | 41 | (66%) | 8 | (18%) | 23 | (55%) | 28 | (74%) | 8 | (31%) | 13 | (54%) | 13 | (54%) | - | (0%) | 9 | (56%) |
| Convulsions/fits | 25 | (40%) | 7 | (16%) | 12 | (30%) | 18 | (47%) | 7 | (27%) | 10 | (42%) | 7 | (29%) | - | (0%) | 2 | (13%) |
| Red eyes with discharge | 9 | (15%) | 5 | (11%) | 1 | (3%) | 6 | (16%) | 5 | (19%) | 1 | (4%) | 3 | (13%) | - | (0%) | - | (0%) |
| Baby feels hot or cold to touch (hypo/hyperthermia) | 26 | (42%) | 4 | (9%) | 8 | (20%) | 16 | (42%) | 4 | (15%) | 2 | (8%) | 10 | (42%) | - | (0%) | 6 | (38%) |
| Vomiting | 9 | (15%) | 4 | (9%) | 4 | (10%) | 6 | (16%) | 4 | (15%) | 4 | (17%) | 3 | (13%) | - | (0%) | - | (0%) |
| Difficulty breathing | 36 | (58%) | 4 | (9%) | 11 | (28%) | 24 | (63%) | 4 | (15%) | 8 | (33%) | 12 | (50%) | - | (0%) | 3 | (19%) |
| Pitched cry and irritability | 32 | (52%) | 2 | (4%) | 16 | (40%) | 22 | (58%) | 2 | (8%) | 9 | (38%) | 10 | (42%) | - | (0%) | 7 | (44%) |
| Unable to pass urine/stool within 24 hours after delivery | 18 | (29%) | 2 | (4%) | 1 | (3%) | 12 | (32%) | 2 | (8%) | - | (0%) | 6 | (25%) | - | (0%) | 1 | (6%) |
| Skin lesions/blisters | 12 | (19%) | 1 | (2%) | 1 | (3%) | 9 | (24%) | 1 | (4%) | - | (0%) | 3 | (13%) | - | (0%) | 1 | (6%) |
| Lethargy/ limpness/ always sleeping | 5 | (8%) | 1 | (2%) | 1 | (3%) | 2 | (5%) | 1 | (4%) | - | (0%) | 3 | (13%) | - | (0%) | 1 | (6%) |
| Blueness of lips, hands or skin | 11 | (18%) | - | (0%) | - | (0%) | 7 | (18%) | - | (0%) | - | (0%) | 4 | (17%) | - | (0%) | - | (0%) |
| Low birth weight, including prematurity | 2 | (3%) | - | (0%) | 1 | (3%) | - | (0%) | - | (0%) | 1 | (4%) | 2 | (8%) | - | (0%) | - | (0%) |
| **Essential Newborn Care** |  |  | - |  | - |  | - |  | - |  | - |  | - |  | - |  | - |  |
| Importance of immunizations | 39 | (63%) | 23 | (51%) | 17 | (41%) | 24 | (63%) | 13 | (50%) | 9 | (38%) | 15 | (63%) | 10 | (53%) | 8 | (47%) |
| Drying and wrapping | 42 | (68%) | 6 | (13%) | 27 | (66%) | 22 | (58%) | 4 | (15%) | 17 | (71%) | 20 | (83%) | 2 | (11%) | 9 | (59%) |
| Advises mother to not apply anything to the cord | 42 | (68%) | 5 | (11%) | 7 | (17%) | 27 | (71%) | 4 | (15%) | 4 | (17%) | 15 | (63%) | 1 | (5%) | 3 | (18%) |
| Delayed bathing | 15 | (24%) | - | (0%) | 9 | (22%) | 10 | (26%) | - | (0%) | 5 | (21%) | 5 | (21%) | - | (0%) | 4 | (24%) |
| Skin-to-skin care method (for normal birthweight babies) | 9 | (15%) | - | (0%) | 2 | (5%) | 5 | (13%) | - | (0%) | 2 | (8%) | 4 | (17%) | - | (0%) | - | (0%) |
| **Care for premature and/or low birth weight newborn** | 0 |  | - |  | - |  | - |  | - |  | - |  | - |  | - |  | - |  |
| Breast milk is the best food for small baby for good weight gain | 35 | (56%) | 2 | (4%) | 3 | (8%) | 18 | (47%) | - | (0%) | - | (0%) | 17 | (71%) | 2 | (11%) | 3 | (19%) |
| Breastfeed more frequently, every 2-3 hours day and night | 45 | (73%) | 2 | (4%) | 4 | (10%) | 27 | (71%) | - | (0%) | 3 | (13%) | 18 | (75%) | 2 | (11%) | 1 | (6%) |
| Keep the small baby warm by Kangaroo Mother Care | 29 | (47%) | - | (0%) | 4 | (10%) | 20 | (53%) | - | (0%) | 3 | (13%) | 9 | (38%) | - | (0%) | 1 | (6%) |
| If the baby is not yet suckling well and long enough, you should express breast milk and feed the baby by cup | 35 | (56%) | - | (0%) | 5 | (13%) | 21 | (55%) | - | (0%) | 1 | (4%) | 14 | (58%) | - | (0%) | 1 | (6%) |
| **Infant feeding and nutrition** | 0 |  | - |  | - |  | - |  | - |  | - |  | - |  | - |  | - |  |
| Advises the mother to exclusively breastfeed | 45 | (73%) | 21 | (47%) | 10 | (24%) | 27 | (71%) | 16 | (62%) | 4 | (17%) | 18 | (75%) | 5 | (26%) | 6 | (35%) |
| Discuss/demonstrate how to breastfeed | 20 | (32%) | 21 | (47%) | - | - | 11 | (29%) | 17 | (65%) | - | - | 9 | (38%) | 4 | (21%) | - | - |
| Complementary feeding | 49 | (79%) | 13 | (29%) | 23 | (56%) | 31 | (82%) | 8 | (31%) | 13 | (54%) | 18 | (75%) | 5 | (26%) | 9 | (59%) |
| Mentions that breastmilk contains adequate water and nutrients | 42 | (68%) | 12 | (27%) | 24 | (59%) | 23 | (61%) | 6 | (23%) | 14 | (58%) | 19 | (79%) | 6 | (32%) | 9 | (59%) |
| To breastfeed on demand (feed 8-10 times per day) | 52 | (84%) | 5 | (11%) | 13 | (32%) | 31 | (82%) | 5 | (19%) | 7 | (29%) | 21 | (88%) | - | (0%) | 6 | (35%) |
| Mentions that breastfeeding promotes mother-baby bonding | 14 | (23%) | 1 | (2%) | 2 | (5%) | 8 | (21%) | 1 | (4%) | 2 | (8%) | 6 | (25%) | - | (0%) | - | (0%) |
| Breastfeeding within the first hour of birth | 41 | (66%) | - | (0%) | 7 | (17%) | 26 | (68%) | - | (0%) | 3 | (13%) | 15 | (63%) | - | (0%) | 4 | (24%) |
| Breastfeeding first yellowish milk (colostrum) | 31 | (50%) | - | (0%) | 7 | (17%) | 19 | (50%) | - | (0%) | 5 | (21%) | 12 | (50%) | - | (0%) | 2 | (12%) |
| **Family planning** |  |  |  |  |  |  |  |  |  |  |  |  |  |  |  |  |  |  |
| Talks about benefits of family planning | 57 | (92%) | 30 | (67%) | 30 | (73%) | 35 | (92%) | 17 | (65%) | 15 | (63%) | 22 | (92%) | 13 | (68%) | 14 | (88%) |
| Discusses return to fertility | 10 | (16%) | 8 | (18%) | 8 | (20%) | 6 | (16%) | 1 | (4%) | 5 | (21%) | 4 | (17%) | 7 | (37%) | 3 | (19%) |
| Birth spacing: Discusses waiting two years before having the next pregnancy | 9 | (15%) | 6 | (13%) | 30 | (73%) | 4 | (11%) | - | (0%) | - | (0%) | 5 | (21%) | 6 | (32%) | 1 | (6%) |
| Talks about partner involvement | 21 | (34%) | 6 | (13%) | 7 | (18%) | 14 | (37%) | 6 | (23%) | 4 | (17%) | 7 | (29%) | - | (0%) | 3 | (19%) |
| Discusses where to obtain family planning methods | 40 | (65%) | 26 | (58%) | 27 | (67%) | 23 | (61%) | 14 | (54%) | 15 | (63%) | 17 | (71%) | 12 | (63%) | 12 | (73%) |
| Discusses range of family planning methods | 53 | (85%) | 14 | (31%) | 30 | (73%) | 33 | (87%) | 6 | (23%) | 15 | (63%) | 20 | (83%) | 8 | (42%) | 11 | (69%) |
| Discusses LAM | 20 | (32%) | 7 | (16%) | 2 | (5%) | 18 | (47%) | 7 | (27%) | 1 | (4%) | 2 | (8%) | - | (0%) | 1 | (6%) |
| *Mentions LAM criteria of menses not returned* | 42 | (68%) | 6 | *(13%)* | 1 | (3%) | 29 | (77%) | 6 | *(23%)* | 1 | (4%) | 13 | (55%) | - | *(0%)* | - | (0%) |
| *Mentions LAM criteria of breastfeeding day and night* | 53 | (86%) | 3 | *(7%)* | 1 | (3%) | 34 | (90%) | 3 | *(12%)* | 1 | (4%) | 1 | (5%) | - | *(0%)* | - | (0%) |
| *Mentions LAM criteria of baby being less than 6 months* | 36 | (58%) | 5 | *(11%)* | 2 | (5%) | 29 | (77%) | 5 | *(19%)* | 1 | (4%) | 7 | (30%) | - | *(0%)* | 1 | (6%) |
| **HIV/ AIDS** |  |  |  |  |  |  |  |  |  |  |  |  |  |  |  |  |  |  |
| *Modes of transmission from one person to another:* | 0 |  | - |  | - |  | - |  | - |  | - |  | - |  | - |  | - |  |
| Through infected mother to her child | 47 | (76%) | 4 | (9%) | 8 | (20%) | 31 | (82%) | 3 | (12%) | 5 | (21%) | 16 | (67%) | 1 | (5%) | 3 | (19%) |
| Through sexual intercourse with infected person | 61 | (98%) | - | (0%) | 37 | (90%) | 38 | (100%) | - | (0%) | 20 | (83%) | 23 | (96%) | - | (0%) | 16 | (100%) |
| Through blood transfusion | 42 | (68%) | - | (0%) | 6 | (15%) | 23 | (61%) | - | (0%) | 4 | (17%) | 19 | (79%) | - | (0%) | 2 | (13%) |
| Through contaminated medical equipment and/or sharp objects | 52 | (84%) | - | (0%) | 24 | (58%) | 30 | (79%) | - | (0%) | 11 | (46%) | 22 | (92%) | - | (0%) | 12 | (75%) |
| *Prevention of HIV/AIDS transmission* | 0 |  | - |  | - |  | - |  | - |  | - |  | - |  | - |  | - |  |
| Condom use | 48 | (77%) | 1 | (2%) | 25 | (60%) | 32 | (84%) | 1 | (4%) | 16 | (67%) | 16 | (67%) | - | (0%) | 8 | (50%) |
| Abstinence | 19 | (31%) | - | (0%) | 5 | (13%) | 12 | (32%) | - | (0%) | 3 | (13%) | 7 | (29%) | - | (0%) | 2 | (13%) |
| Being monogamous/faithful to one partner | 48 | (77%) | - | (0%) | 26 | (63%) | 29 | (76%) | - | (0%) | 12 | (50%) | 19 | (79%) | - | (0%) | 13 | (81%) |
| Avoids sharing needles, razors or sharp objects | 37 | (60%) | - | (0%) | 18 | (45%) | 23 | (61%) | - | (0%) | 8 | (33%) | 14 | (58%) | - | (0%) | 10 | (63%) |
| *Modes of mother to child HIV transmission* | 0 |  | - |  | - |  | - |  | - |  | - |  | - |  | - |  | - |  |
| After delivery, through breastfeeding | 55 | (89%) | 2 | (4%) | 27 | (65%) | 35 | (92%) | 1 | (4%) | 14 | (58%) | 20 | (83%) | 1 | (5%) | 12 | (75%) |
| During pregnancy (in utero) | 46 | (74%) | 1 | (2%) | 18 | (43%) | 28 | (74%) | - | (0%) | 15 | (63%) | 18 | (75%) | 1 | (5%) | 2 | (13%) |
| During delivery | 54 | (87%) | 1 | (2%) | 14 | (35%) | 34 | (89%) | - | (0%) | 10 | (42%) | 20 | (83%) | 1 | (5%) | 4 | (25%) |
| *Preventing mother to child HIV transmission* | 0 |  | - |  | - |  | - |  | - |  | - |  | - |  | - |  | - |  |
| ARVs | 53 | (85%) | 3 | (7%) | 25 | (60%) | 34 | (89%) | 2 | (8%) | 15 | (63%) | 19 | (79%) | 1 | (5%) | 9 | (56%) |
| Exclusive breastfeeding | 54 | (87%) | 1 | (2%) | 8 | (20%) | 32 | (84%) | - | (0%) | 6 | (25%) | 22 | (92%) | 1 | (5%) | 2 | (13%) |
| Non-breastfeeding | 25 | (40%) | 1 | (2%) | 6 | (15%) | 15 | (39%) | - | (0%) | 2 | (8%) | 10 | (42%) | 1 | (5%) | 4 | (25%) |
| *HIV Testing* | 0 |  | - |  | - |  | - |  | - |  | - |  | - |  | - |  | - |  |
| Asks the mother if she knows her HIV status | 45 | (73%) | 2 | (4%) | 23 | (55%) | 30 | (79%) | 2 | (8%) | 12 | (50%) | 15 | (63%) | - | (0%) | 10 | (63%) |
| Encourages the mother to get tested for HIV | 57 | (92%) | 3 | (7%) | 30 | (72%) | 34 | (89%) | 3 | (12%) | 14 | (57%) | 23 | (96%) | - | (0%) | 15 | (94%) |
| **Malaria** |  |  |  |  |  |  |  |  |  |  |  |  |  |  |  |  |  |  |
| Encourages mother to have baby sleep under bed net | 61 | (98%) | 6 | (13%) | 25 | (62%) | 37 | (97%) | 6 | (23%) | 17 | (70%) | 24 | (100%) | - | (0%) | 8 | (50%) |
| Provides ITN voucher | 58 | (94%) | - | (0%) | 21 | (51%) | 37 | (97%) | - | (0%) | 10 | (43%) | 21 | (88%) | - | (0%) | 10 | (63%) |
| Tell woman where to obtain an ITN | 39 | (63%) | - | (0%) | - | - | 24 | (63%) | - | (0%) | - | - | 15 | (63%) | - | (0%) | - | - |

* Minimum content recommended by WHO, *WHO recommendations on Postnatal care of the mother and newborn*. 2013, World Health Organization, Department of Maternal, Newborn, Child and Adolescent Health: Geneva, Switzerland. In instances where an * is noted on a domain heading (e.g. maternal nutrition) the sub-content has not been explicitly defined by WHO.
